# 559 Verticalization Therapy for ICU-Level Burn Patient: A Case Study

**DOI:** 10.1093/jbcr/irad045.155

**Published:** 2023-05-15

**Authors:** Melinda Tepe, Nicole Gerlich, Allison O’Donnell, Mack Drake, Michael Feldman

**Affiliations:** VCU Health System, Mechanicsville, Virginia; VCU Health System, Henrico, Virginia; Virginia Commonwealth University Health System, Mechanicsville, Virginia; VCU Health System, Richmond, Virginia; VCU Health System, Richmond, Virginia; VCU Health System, Mechanicsville, Virginia; VCU Health System, Henrico, Virginia; Virginia Commonwealth University Health System, Mechanicsville, Virginia; VCU Health System, Richmond, Virginia; VCU Health System, Richmond, Virginia; VCU Health System, Mechanicsville, Virginia; VCU Health System, Henrico, Virginia; Virginia Commonwealth University Health System, Mechanicsville, Virginia; VCU Health System, Richmond, Virginia; VCU Health System, Richmond, Virginia; VCU Health System, Mechanicsville, Virginia; VCU Health System, Henrico, Virginia; Virginia Commonwealth University Health System, Mechanicsville, Virginia; VCU Health System, Richmond, Virginia; VCU Health System, Richmond, Virginia; VCU Health System, Mechanicsville, Virginia; VCU Health System, Henrico, Virginia; Virginia Commonwealth University Health System, Mechanicsville, Virginia; VCU Health System, Richmond, Virginia; VCU Health System, Richmond, Virginia

## Abstract

**Introduction:**

The entire interdisciplinary team can be faced with many challenges when treating a bariatric, critically ill patient with significant burn injuries. Some studies suggest that the care of these patients increases staff workload and leads to the need for increased assistance for proper turning, positioning, and completion of wound care. When caring for these types of patients, patient and staff safety should be considered, as well as what will ensure the best patient outcomes. Our rehab team initiated the use of verticalization therapy with a patient on the burn unit with 28% TBSA full-thickness burns to back, chest, BUEs and a BMI of 45.8 in order to promote improved healing, cognitive status, and pulmonary function. While verticalization therapy is described for use in patients with pulmonary issues or invasive cardiac support devices, this was the first time this therapy was used in our busy ABA-verified burn center.

**Methods:**

Once deemed appropriate for participation with PT/OT, a collaborative protocol with nursing, therapy, and burn provider team was developed. The patient was transitioned into a specialized bed allowing for verticalization up to 90 degrees. The patient was incrementally verticalized by 10 degrees at a time while monitoring vitals, assessing patient safety and pain response. Ultimately, the patient reached 40 degrees vertical and maintained this position for up to 2 hours. This intervention was utilized by both rehab specialists and nursing staff and was taught to nursing staff to be performed daily until patient was able to actively participate and tolerate edge of bed or out of bed activity.

**Results:**

Use of the verticalization positioning was used to promote weight bearing activity, improve pulmonary function, improve cognitive status through upright positioning, and for pressure relief of graft areas specifically for offloading pressure to back wounds and grafts. The bed was used for 1 month. During this time, the patient successfully liberated from mechanical ventilation and shock state and mobilized with therapy assistance. The patient demonstrated improved strength, active participation with therapies, and decreased agitation as the month progressed. Autograft split-thickness skin grafts with autologous cell suspension epithelial autografts to the patient’s posterior torso healed well and were fully closed by the time of discharge to inpatient rehabilitation.

**Conclusions:**

Use of verticalization beds can be implemented in the care of patients with burn injuries safely and successfully. Further research regarding skin healing related to use of the bed and verticalization therapy in burn injured patients should be conducted.

**Applicability of Research to Practice:**

This intervention can be used for patients of all sizes and can be implemented as soon as hospital day one, if the patient is medically appropriate

